# Application of unsupervised clustering model based on graph embedding in water environment

**DOI:** 10.1038/s41598-023-50301-2

**Published:** 2023-12-20

**Authors:** Meng Fang, Li Lyu, Ning Wang, Xiaolei Zhou, Yankun Hu

**Affiliations:** 1 University of Chinese Academy of Sciences, Beijing, China; 2 Shenyang Institute of Computing Technology, Chinese Academy of Sciences, Shenyang, China

**Keywords:** Computer science, Information technology

## Abstract

Surface water monitoring data has spatiotemporal characteristics, and water quality will change with time and space in different seasons and climates. Data of this nature brings challenges to clustering, especially in terms of obtaining the temporal and spatial characteristics of the data. Therefore, this paper proposes an improved TADW algorithm and names it RTADW to obtain the spatiotemporal characteristics of surface water monitoring points. We improve the feature matrix in TADW and input the original time series data and spatial information into the improved model to obtain the spatiotemporal feature vector. When the improved TADW model captures watershed information for clustering, it can simultaneously extract the temporal and spatial characteristics of surface water compared with other clustering algorithms such as the DTW algorithm. We applied the proposed method to multiple different monitoring sites in the Liaohe River Basin, analyzed the spatiotemporal regional distribution of surface water monitoring points. The results show that the improved feature extraction method can better capture the spatiotemporal feature information between surface water monitoring points. Therefore, this method can provide more potential information for cluster analysis of water environment monitoring, thereby providing a scientific basis for watershed zoning management.

## Introduction

In recent years, with the introduction of the concept of smart environmental protection, the continuous development of new sensing technology and intelligent management technology of ecological environment, the importance of scientific governance of water ecological environment has increased dramatically. In particular, excessive discharge of various pollutants exceeds natural self-purification capabilities, causing changes in the water environment, destroying ecological balance, and causing harm to the human body, production and life. Therefore, surface water pollution remains a global problem^1,2^. To prevent pollution of ecological watersheds caused by agriculture, industry, and daily life, it is necessary to regularly monitor the quality of surface water to scientifically manage the water ecological environment. An important component of water ecological management is the zoning management of watersheds^3^.

The watershed monitoring points are interconnected by several mainstream tributaries, so the water quality data between the monitoring points have both temporal and spatial characteristics^4^. Realize the regional division of water ecological watersheds and analyze the spatiotemporal characteristics of monitoring points at different spatial locations, so that the flow sections of each region can be managed more scientifically. By performing spatiotemporal analysis of water quality data between monitoring points to form clusters with similar characteristics, the periodicity of water quality changes between different monitoring points can be found. Since the correlation between monitored water quality indicators cannot be directly described^4–6^, statistical mathematical methods and models are used to analyze water quality parameters and explore the hidden information between multi-dimensional water quality data at monitoring points.

Statistical mathematical methods are used to analyze water quality-related parameters. Commonly used methods mainly include linear multivariate statistical methods, principal component analysis and cluster analysis techniques^4,5,7^. Fuzzy logic, as a variant of statistical mathematical methods, is also used in research^4^. Linear multivariate statistical methods, such as principal component analysis, discriminant analysis and cluster analysis, have also achieved certain results in most past studies^8,9^. As a technical means, cluster analysis has been widely used in atmospheric and water environment assessment and functional zoning research. Deng Zheng-yu et al. used K-means clustering algorithm to achieve clustering of ecosystem clusters and regional planning of functional areas in the Shennongjia area^10^. Lee et al. proposed a time series water quality data clustering method based on dynamic time programming. They used Euclidean distance and DTW algorithm to perform cluster analysis on 12 sites respectively, and also used CVI method to determine the optimal number of clusters and obtain a classification result map of water quality data^11^. Tahir Ali et al. used principal component analysis and clustering methods to cluster 18 lakes. Principal component analysis was used to determine the main water quality parameters, and pattern matching was used to identify clusters for each lake^4^. Li et al. proposed a comprehensive model based on K-means cluster analysis and set pair analysis, applied the model to the reservoir area to conduct water pollution risk assessment, and realized the use of cluster analysis method to determine the source of pollution^12^. Jatnika et al. applied the k-means method to the mapping of water pollution areas in Indonesia, and passed the analysis results to government departments to focus on the management of water pollution areas^13^. Birant et al. proposed a new density-based clustering algorithm ST-DBSCAN and applied it to ocean data to explore areas with similar seawater characteristics^14^. Mosavi et al. used the FCM method to identify donor basins similar to ungauged basins, using fuzzy membership to determine the membership of each basin^15^.

By analyzing the above research methods for water environment assessment and functional zoning, it can be seen that these linear statistical methods and cluster analysis methods have achieved certain research results in surface water quality assessment and ecological environment zoning management. However, current water quality parameter feature extraction and water environment partition clustering still face the following problems: (1) Linear multivariate statistical methods are easily restricted by linear assumptions, and linear statistical methods have limitations in extracting spatial features of data^16^. (2) The DTW algorithm is widely used in time series clustering^17^, but water environment partitioning that only considers temporal characteristics will lead to the loss of spatial information about the interaction between some monitoring points, reducing the accuracy of the partitioning. (3) When processing multi-attribute spatiotemporal data, clustering methods cannot obtain the potential characteristics and multi-dimensional relationships of the data, and existing methods require manual setting of multiple parameters.

To solve the problems of the above clustering algorithm in water environment analysis and improve the accuracy of watershed partitioning, we proposed a network embedded clustering analysis method that combines the network structure information of watershed monitoring points and the multi-attribute characteristics of monitoring points. In the watershed monitoring network, each monitoring point has a set of time-characteristic water quality indicator information, such as chemical oxygen demand, total phosphorus, total nitrogen, etc., and these characteristics will interact in the watershed. The algorithm uses a network embedding method to simultaneously capture the structural information and attribute information similarities between watershed monitoring stations and construct related spatiotemporal feature vectors. More specifically, we first obtain water quality time series data at different monitoring points in the watershed, as well as the spatial location information of the stations. Then the attribute feature matrix of different monitoring points and the adjacency matrix of monitoring sites are constructed. Then we improve the feature matrix T in the TADW algorithm and use the cosine similarity of the monitoring stations to perform fusion calculations on the feature matrix T to obtain a vector representation of the similarity between different monitoring stations. Then the improved TADW algorithm is used to obtain the embedding vectors of the watershed monitoring points, thereby obtaining the spatiotemporal feature vector matrix of the clustering input. In this paper, our contributions can be summarized as follows:

1. We use the improved TADW algorithm to obtain the spatiotemporal characteristic information between different monitoring points in the watershed, named it RTADW, to better extract potential information in water environment monitoring water quality.
2. We have improved the input vector of the cluster analysis algorithm, which contains both temporal information and spatial information of multi-attribute data points.
3. The proposed method was tested on data sets from different monitoring points in a real watershed and compared with other watershed partitioning methods, verifying that our algorithm has better accuracy in watershed partitioning.

## Related work

In this section, we first introduce the feature extraction and community detection methods on network structure data, and then discuss the part on the water environment network partition evaluation method. Generally speaking, network structure feature extraction is mainly based on deep learning methods, among which the graph embedding method is the most commonly used method. Common graph embedding methods are mainly divided into shallow graph representation and deep graph representation learning. The shallow graph embedding model mainly generates multiple node lists through a random walk strategy, and then uses the skip gram model to train each node vector. The main methods include DeepWalk^18^, Node2Vec^19^, and Metapath2vec^20^. The depth graph embedding model refers to combining the graph with the depth model to achieve end-to-end training model. Thus, the spatial structure features of the topological graph are extracted from the graph. Common neural network methods include GCN^21^, GraphSAGE^22^, GAT^23^, etc. Community detection is to use a Euclidean space clustering method, such as k-means^24^, on the data obtained after graph embedding to identify the clusters of the graph^25,26^.

In the remainder of this section, we briefly introduce previous research in the field of water environment zoning. In the past few years, different algorithms for water quality zoning or ecological function zoning have been proposed. We will introduce some of these methods in this section.

Generally speaking, there are three main categories of water environment zoning methods: mathematical statistics-based methods, model-based methods and time series analysis-based clustering methods. By analyzing water environment monitoring indicators based on mathematical statistics methods, classification or discrimination is made by analyzing the linear correlation between indicators. These methods mainly include multivariate linear analysis method, principal component analysis method, discriminant analysis and cluster analysis method^7,27–30^. Among them, the most famous cluster analysis method is the k-means method, which divides the data set through continuous iterative calculations so that the distance between data in the same cluster is close and the distance between different clusters is far. The model-based method consists in establishing relevant models, mining potential information on water quality indicators, and then conducting cluster analysis on them. Model-based methods are further divided into two categories: water quality model-based and neural network model-based^31–34^. The former is based on water quality indicators for index calculation in the field of water environment science. The steps mainly include: (1) determine relevant pollutants based on calculation formulas in the field of environmental science; (2) substitute pollutants, flow rates and other indicators into the formula to calculate relevant values; (3) establish a water quality model. The latter is to establish a neural network model to extract features of water environment monitoring indicators, and then perform cluster analysis. Based on the time series cluster analysis method, the most famous method is the DTW algorithm^11^. Its main steps include: (1) calculate the DTW distance vector matrix between monitoring indicators at different points. (2) Construct a similarity matrix. (3) Perform cluster analysis or visualization.

## Our methods

### Study area

**Figure 1:**
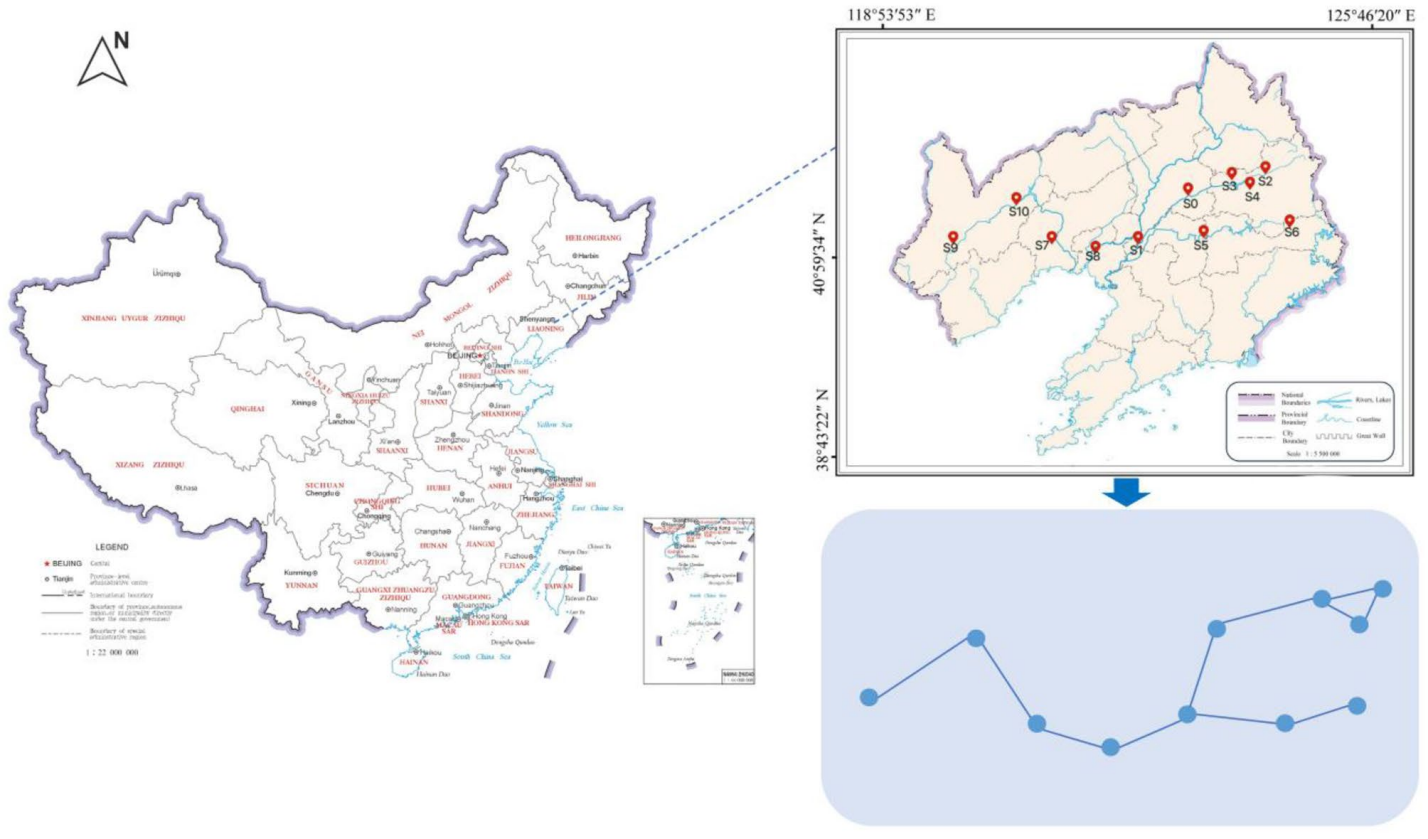
Location information of monitoring stations in the Liaohe River Basin.

The Liaohe River Basin is located in the southwest of Northeast China. The entire basin is composed of the Liaohe River System and the Daliaohe River System. The Liao River has a total length of 1390 km and a drainage area of 219,000 km^2^. It is formed by the confluence of the East Liao River, the West Liao River and other tributaries in Liaoning Province. The main stream of the river is mainly in Liaoning Province. The Liaohe River Basin in Liaoning Province covers 8 provincial cities including Tieling, Shenyang and Anshan. The spatial distribution of monitoring points in the Liaohe River Basin and its representation simplified into a network diagram are shown in Fig. 1.

### Data

#### Monitoring data characteristics

To study the temporal and spatial characteristics of water pollution, in this study we used the monthly data of 11 monitoring stations in the Liaohe River Basin from 2018 to 2022 for analysis. The monitoring stations include 6 main stations (Zhaoquan River-S8, Yujiafang-S1, Dongling Bridge-S0, Taigou-S3, Beizamu-S2, Zhangjiying-S10) and 5 tributary stations (Xing'an-S5, Beitaizi River-S6, Gulou-S4, Xishulin-S7, Wang's Shack-S9). The pollution indicators for data analysis include hydrogen ion concentration index, ammonia nitrogen, permanganate index, dissolved oxygen, water temperature, total phosphorus, and total nitrogen.

The basic statistics of water pollution indicators are shown in Table 1. The mean interpolation method is a common method for supplementing numerical missing values. Some sites have missing data due to monitoring equipment or Internet of Things failures, but our number of missing data is less than 10%. To ensure the continuity of the data, the mean interpolation method is used to complete the missing data of the water quality data. Since the Liaohe River Basin is located in the northeastern region of China, some monitoring stations will encounter a large number of missing data collection in winter, so the data we use is the real-time monitoring monthly data from January to October.

**Table 1:** Descriptive statistics of the monitoring data.

Variable	Description	Statistical	S0	S1	S2	S3	S4	S5	S6	S7	S8	S9	S10
PH	Potential of Hydrogen	Mean	7.92	7.639	7.566	7.604	7.69	7.875	7.703	8.118	7.872	7.915	8.084
		S.D.	0.308	0.328	0.437	0.334	0.336	0.387	0.369	0.298	0.254	0.289	0.216
AN (mg/L)	Ammonia nitrogen	Mean	0.809	0.737	0.2	0.119	0.148	0.412	0.136	0.806	0.718	0.25	0.42
		S.D.	1.014	1.259	0.179	0.176	0.178	0.502	0.162	1.276	0.852	0.163	0.381
PI (mg/L)	Permanganate index	Mean	4.578	5.853	2.924	2.167	2.713	3.108	2.347	4.931	6.657	2.939	4.064
		S.D.	1.432	1.632	1.119	1.058	0.964	0.987	1.156	2.077	3.117	0.661	1.608
DO (mg/L)	Dissolved Oxygen	Mean	8.89	7.97	9.364	8.953	9.401	8.16	9.169	9.486	9.077	9.472	9.556
		S.D.	2.29	2.058	1.704	1.402	1.531	1.992	1.768	2.868	3.577	1.68	1.571
WT (°C)	Water temperature	Mean	14.369	11.929	13.826	13.428	12.654	13.557	14.07	13.588	13.205	13.066	14.003
		S.D.	7.653	5.4	7.312	7.518	7.19	6.337	5.576	7.685	7.745	6.996	8.763
TN (mg/L)	Total nitrogen	Mean	6.388	6.451	5.826	5.682	6.197	6.18	5.617	7.847	6.632	7.459	7.397
		S.D.	1.697	1.727	1.883	1.965	1.683	1.86	1.989	2.071	1.624	2.036	2.391
TP (mg/L)	Total phosphorus	Mean	0.719	0.759	0.178	0.116	0.138	0.512	0.131	0.71	0.687	0.196	0.344
		S.D.	1.043	1.245	0.160	0.153	0.157	0.346	0.142	1.305	0.864	0.168	0.378

### Algorithm design

Before introducing the algorithm, first introduce the basic conceptual information, which is the basis of the algorithm.

#### Concept description

In this study, we focus on watershed monitoring networks with relevant monitoring characteristics. The monitoring site with attributes is defined by $G=(V,E,F$), where $V$ represents the monitoring site, $E$ represents the monitoring site-monitoring site interaction set of edges, and $F$ represents the feature vector set. The total number of vertices is $n=\left|V\right|$, and the total number of edges is $m=\left|E\right|$. To describe the characteristics of a vertex, we represent it as an attribute vector $F=\left({f}_{1},{f}_{2},{f}_{3}\dots {f}_{L}\right)$, where $L$ is the number of features.

#### Embedding the information of both structure and feature

In order to be able to perform the task of water environment partitioning in a watershed monitoring water quality characteristics network, two data sources in the monitoring network can be used. The spatial watershed network formed between different monitoring sites provides the first source of data, while the water quality indicator data at the monitoring sites provides the second source of data. In the watershed network where different monitoring stations interact, there are more and more data of rich graph structure types such as pollutant indicators. In order to extract spatiotemporal features in a watershed network, it is more important than ever to simultaneously consider the structure and characteristic attribute information of the watershed network graph. The relationship between nodes with similar characteristics is greater than the relationship between nodes with different characteristics. Therefore, the characteristic information between monitoring points will affect the relationship between different monitoring points^35,36^. Embedded networks are widely recognized as an effective network representation. The essence of network embedding technology is to embed nodes in the network into vectors based on the graph structure and other characteristic attribute information of the network. Combining embedding representation network techniques with clustering methods can provide substantial benefits in finding precise clusters. The representative method in network embedding is DeepWalk^18^, and the TADW method as its extension is used to obtain richer network embedding.

In the current work, we propose to use the attribute embedding method to simultaneously capture the spatial structure and attribute similarity between watershed monitoring sites, that is, to obtain the spatiotemporal similarity matrix embedding vectors of different monitoring sites. More specifically, we propose an improvement to the TADW method, and the improved TADW method is used to obtain the spatial similarity feature embedding vectors of network structures at different monitoring sites.

The TADW^37^ method is a method based on matrix decomposition, which introduces the textual features of nodes into graph representation learning. Its goal is to fuse text features to obtain a representation of the entire network information. The feature $T$ part of the TADW algorithm uses the TF-IDF algorithm to represent text features. In view of the characteristics of water environment monitoring data, we propose to improve the feature $T$ and take the similarity score matrix between objects into the feature extraction task, and name the improved algorithm RTADW. Use the cosine similarity matrix to calculate the external feature $T$ and obtain a new representation feature ${T}{\prime}$. As shown in Formula 1, where T is the original feature matrix, $C$ is the cosine similarity matrix, and the obtained ${T}{\prime}$ is the new feature representation. Then the TADW model framework is used for training, and the improved features ${T}{\prime}$ are input into it. The main step of TADW is to find the minimum value of matrices $W$ and $H$. As shown in Formula (2), $M$ is the decomposition matrix, $T$ is the characteristic matrix, $W$ and $H$ are parameter matrices, and $W$ and $H$ are iteratively updated using gradient descent. In Formula 2, $\Vert \bullet \Vert$ represents the Frobenius norm of the matrix, and λ is the balance factor. Both $W$ and $H$ obtained by TADW can be regarded as representations of vertices. We represent the network by connecting them to form a unified network representation 2k-dimensional matrix^37^. After obtaining the embedding representations of different monitoring points, the subsequent cluster analysis evaluation is performed.

1 $${T}{\prime}=T\times C+T$$

2 $$\underset{W,H}{{\text{min}}}{\Vert M-{W}^{T}HT\Vert }_{F}^{2}+\frac{\lambda }{2}({\Vert W\Vert }_{F}^{2}+{\Vert H\Vert }_{F}^{2})$$

We use TADW for two important key points, which are emphasized as follows: first, to better analyze the data; second, to use a characteristic watershed monitoring network to obtain structural and content similarities between monitoring sites. So, we use TADW to obtain the embedding vector representation of each monitoring site.

#### Propose methods

Assuming the feature graph $G = (V, E, F)$, the purpose of studying the clustering problem in this study is to divide the node set of $G$ into different subsets, so that: (1) The structure and attributes of the monitoring points in the watershed network are similar; (2) The monitoring points of the watershed network contain both structural information and attribute information in terms of characteristic values, and different monitoring points may have different characteristic values.

In previous work, traditional clustering algorithms calculated distances between samples and repeated iterations to finally divide the samples into different clusters. However, in real networks, it is difficult to take the structural information of the network into account.

To solve the clustering problem of multi-feature networks, a new clustering algorithm TADW-C is proposed. The main contribution of this work is the introduction of a new embedding representation matrix to represent the original data, which has both structural and attribute information. To this end, the structural information and characteristic information of the watershed monitoring network are first embedded into vector representations. In the watershed monitoring network, the structure-based network embedding is not enough, and the feature information is highly correlated with the network. Since the external information part of the TADW algorithm is text feature information, the importance of words is measured by word frequency to obtain T. Therefore, this paper improves the feature matrix T in TADW to make the similarity score between monitoring nodes higher. Cosine similarity is a measure of cosine similarity between vectors, and its similarity is expressed as shown in Formula 3, where ${x}_{i}$ and ${y}_{i}$ represent the elements that constitute vector X and vector Y respectively, and ${\text{cos}}(\theta )$ represents the cosine similarity between the two vectors. For the external information in TADW, we apply similarity weights to the original features and fuse the similarity of nodes into the features.

3 $${\text{cos}}(\theta )=\frac{\sum_{i=1}^{n}{x}_{i}\times {y}_{i}}{\sqrt{\sum_{i=1}^{n}{\left({x}_{i}\right)}^{2}}\times \sqrt{\sum_{i=1}^{n}{\left({y}_{i}\right)}^{2}}}$$

Constructing a high-quality similarity matrix enables the proposed algorithm to better divide spatiotemporally similar nodes. After obtaining the embedding representation, in the second step, original feature information is added, which contains the actual information of the network properties and provides data context and interpretability for the representation of nodes. Then in the third step, the T-SNE method is used to retain the similarity relationship between data while reducing dimensionality. This method can effectively capture the structure and model in the data and is suitable for more types of data. In the final step, clustering is applied to the new data space with more useful data.

**Figure 2:**
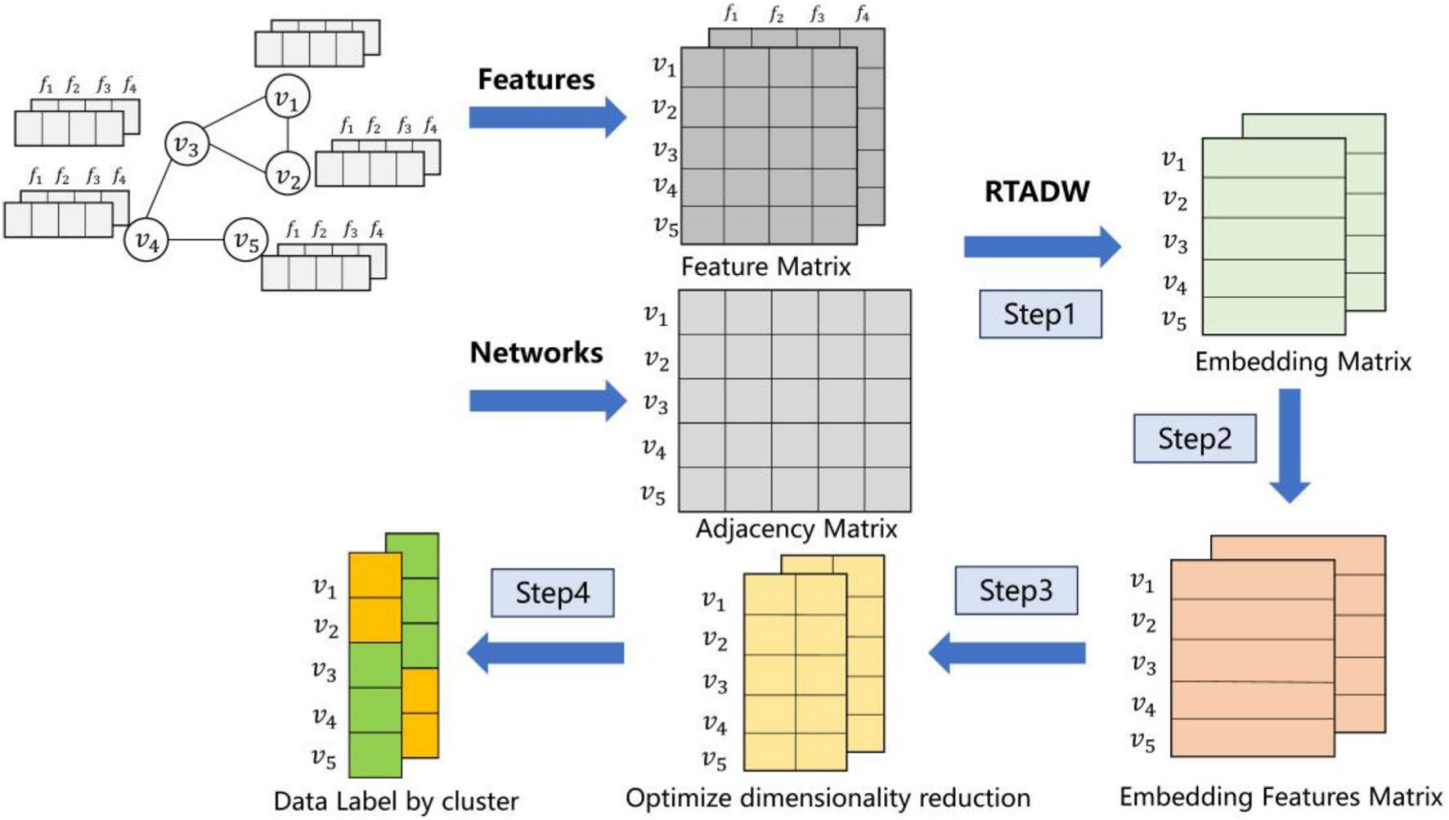
Algorithm Architecture.

A detailed diagram of the algorithm is shown in the Fig. 2. In step 1, the improved TADW is applied to build a high-quality embedding representation matrix. In this process, the spatial structure information and attribute information between monitoring sites are embedded into a new vector space, so that points with similar structural attributes are embedded closer. After obtaining the new embedding representation, in the second step, the embedding vector and the representation vector of the attributes are obtained by adding the original feature attributes. Optimize the dimensionality of the representation vector obtained in the previous step, and divide the new representation into different clusters through step 4 while retaining the relative distance relationship between data points.

## Experiments

### Experimental results

#### K-means algorithm clustering results

**Figure 3:**
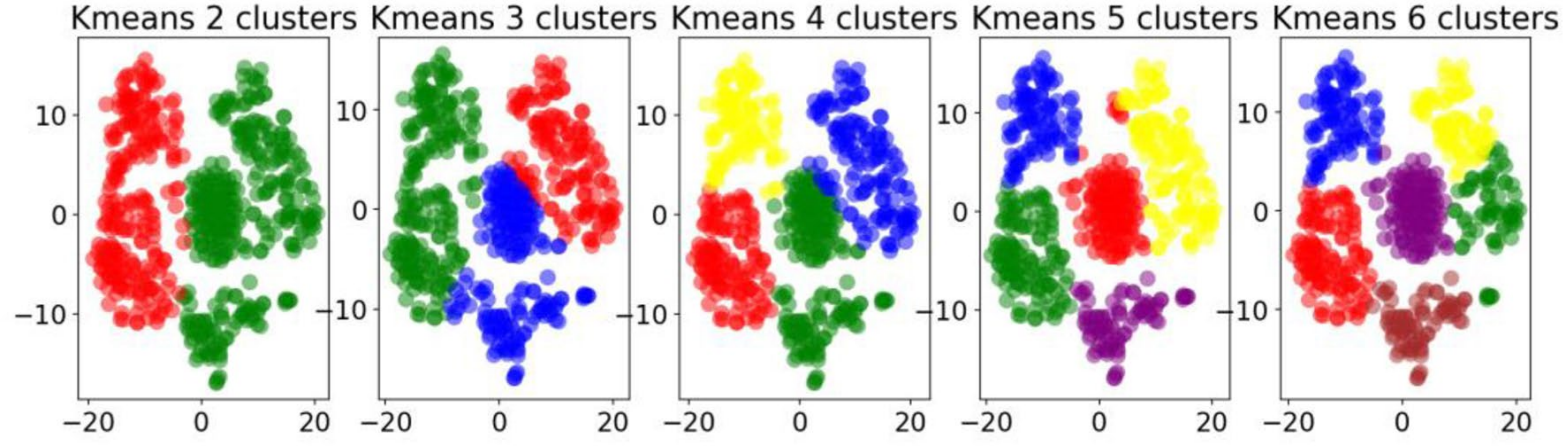
Results of k-means clustering algorithm under different k values.

Since the k-means algorithm requires first setting the number of cluster centers k, the elbow method is a commonly used technique to help determine the k value in k-means clustering. Therefore, we combined the experimental results of the elbow method, set the k value to 2, 3, 4, 5, and 6, and observed the results of k-means respectively. The results are shown in Fig. 3.

**Figure 4:**
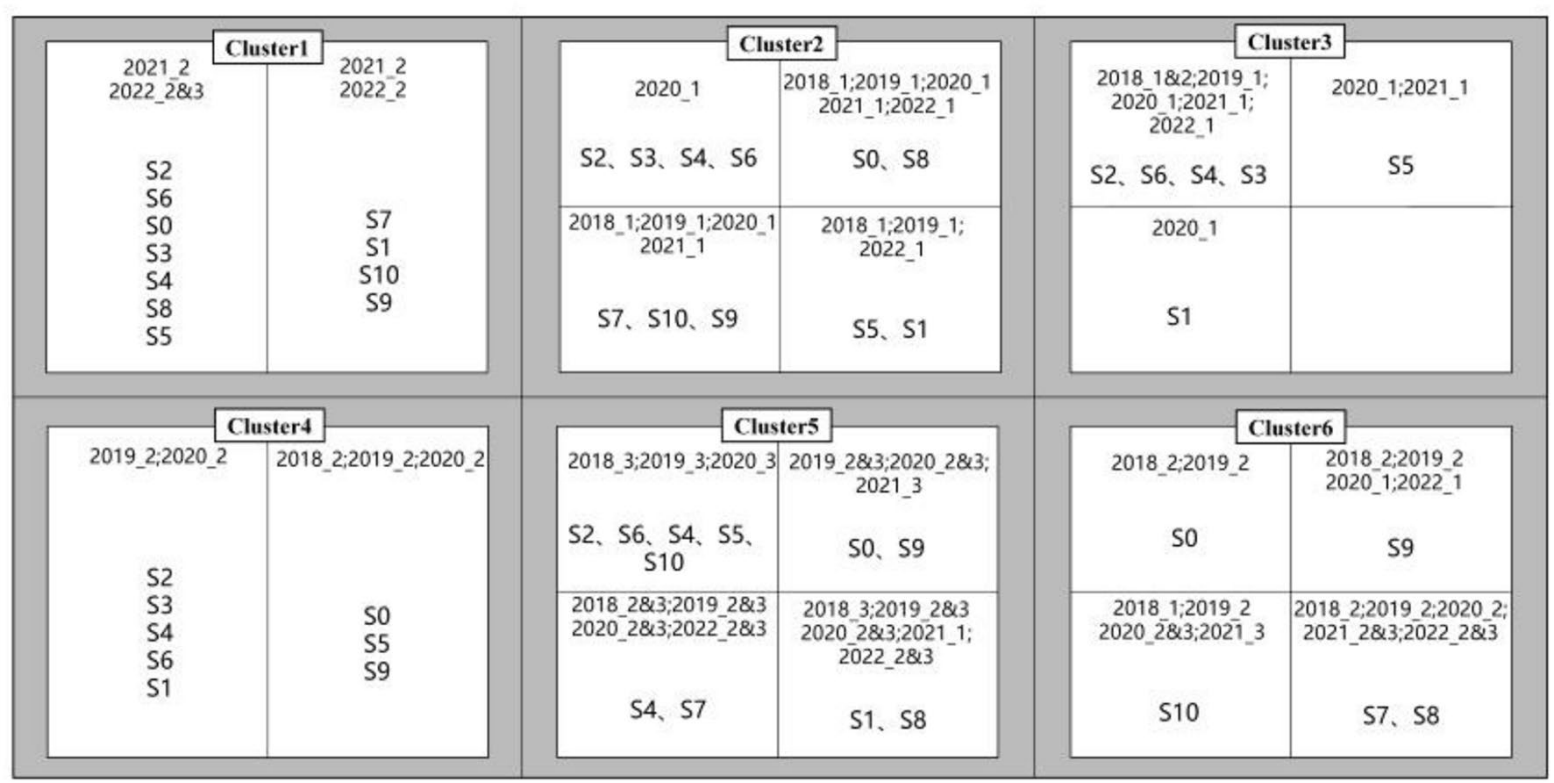
The k-means clustering results obtained by extracting the spatiotemporal characteristics of different monitoring sites through RTADW.

As can be seen from the k-means results in Fig. 3, when k = 6, the boundaries between clusters are clearest, so we believe that the clustering effect is best at this time. By analyzing the clustering results, the spatio-temporal clustering result diagram in Fig. 4 is obtained. It can be seen that in cluster 1, most of the clustering results are data from the second quarter of 2021 and the third quarter of 2022. (October, January and February are defined as the first quarter, March, April and May are defined as the second quarter, and June, July, August and September are defined as the third quarter). From the perspective of time cluster distribution, S2, S6, S0, S3, S4, S8, and S5 are relatively similar in the time range; S7, S1, S10, and S9 are relatively similar. In cluster 2, the clustering results are mostly the data of the first quarter of each year, which can be divided into four time periods from the analysis of the similarity of time periods, as shown in Fig. 4. In cluster 3, most of them are the data distribution of S2, S6, S4, and S3 in the first quarter of each year, and also include part of the second quarter data of S5 and part of the first quarter data of S1. In cluster 4, the results are mostly data from the second quarter of 2018 to 2020. In terms of time distribution, S2, S6, S3, S4, and S1 are relatively similar, and S0, S5, and S9 are relatively similar. In cluster 5, most of the data are from May, June, and October. In terms of time distribution, it can be divided into four time periods, as shown in Fig. 4. In cluster 6, most of the data are from the second and third quarters, but the time distribution is quite different.

#### DBSCAN clustering algorithm results

**Figure 5:**
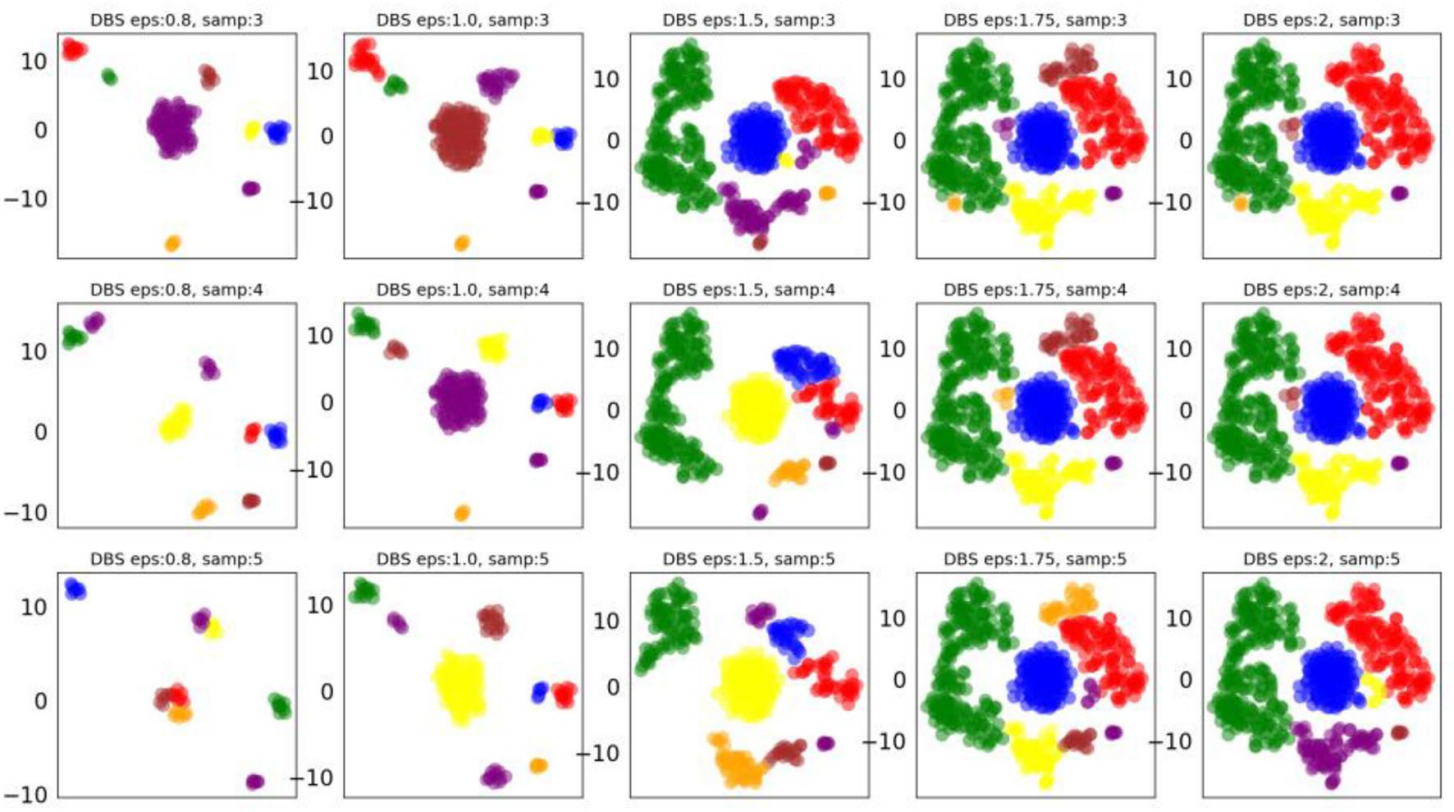
DBSCAN clustering result graph with different parameters.

The DBSCAN algorithm has two parameters that need to be defined: min_samples and eps. We can see the effect of cluster allocation by adjusting the values of the two parameters, as shown in Fig. 5.

**Figure 6:**
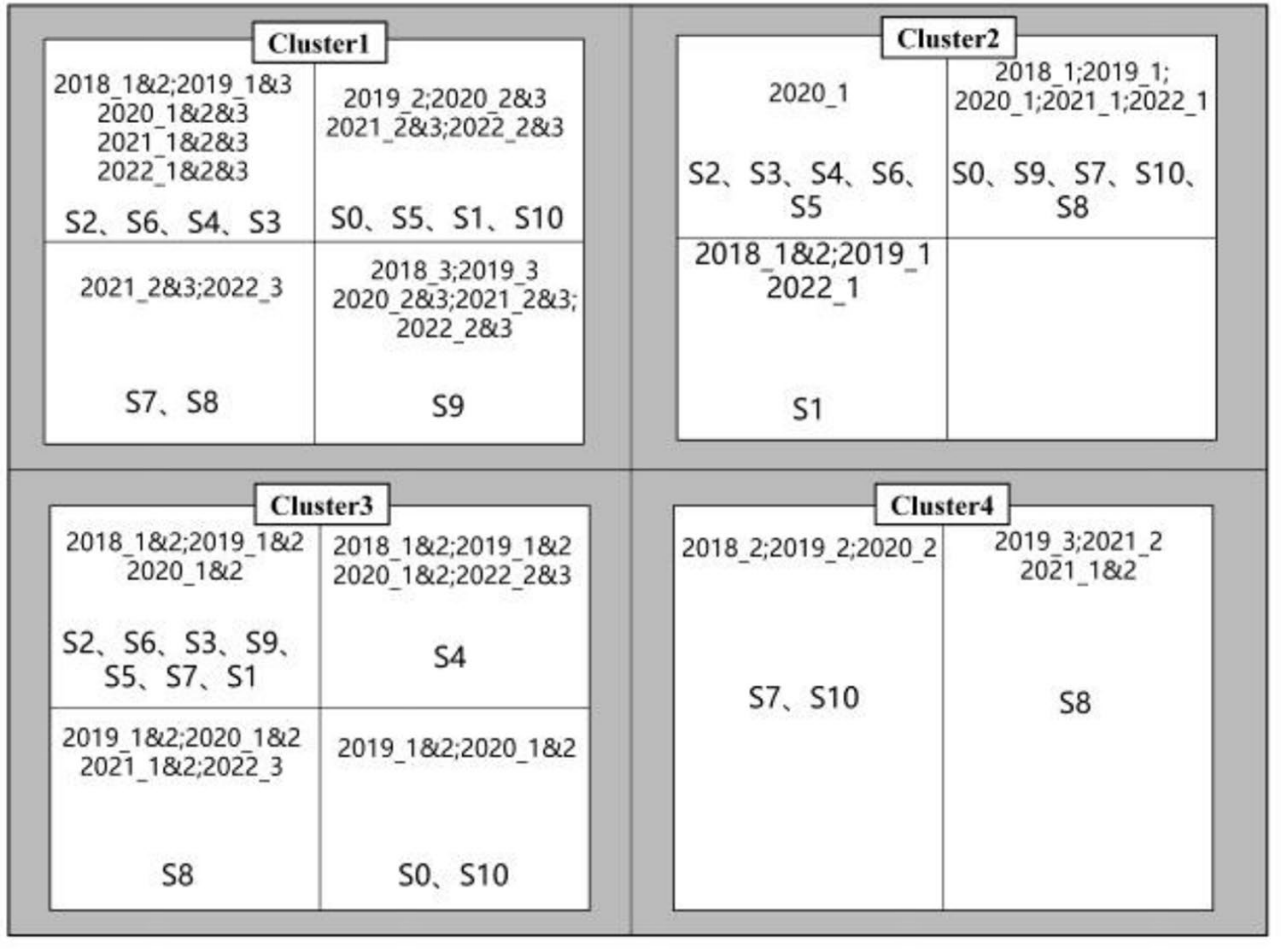
DBSCAN clustering result diagram obtained by extracting spatiotemporal characteristics of different monitoring sites through RTADW.

According to the results in Fig. 5, set the parameters eps = 1.75, min_sample = 4. DBSCAN divides the spatiotemporal data of different watershed monitoring stations into 7 clusters, of which 3 clusters have less than 10 data points and are therefore regarded as outliers. Therefore, we only statistically analyze the results of four clusters, as shown in Fig. 6.

In cluster 1, the cluster distributions of S2, S6, S4, and S3 in the time span are relatively similar, and the data spans from 2018 to 2022. Most of S0, S5, S1, and S10 are the second and third quarters of 2019–2022. S7 and S8 are more similar, and are part of the data for the second and third quarters of 2021–2022. S9 has no similar sites in the time dimension. In cluster 2, S2, S6, S4, S3, and S5 are similar in time dimension, but the amount of data is small. S0, S9, S7, S10, and S8 are more similar, mostly the data distribution of the first quarter of 2018–2022. S1 has no similar stations in the time span. In cluster 3, the monitoring points of S2, S6, S3, S9, S5, S7, and S1 are more similar in time dimension distribution, and most of them are data from May, June, and October from 2018 to 2020. There are no similar points in the time distribution of S4 monitoring points. The S8 site is the data distribution from 2019 to the second and third quarters of 2022. S0 and S10 monitoring points are data from May, June, and October from 2019 to 2020. In cluster 4, only part of the second quarter data of monitoring points S7, S10, and S8 are included.

## Discussion

**Figure 7:**
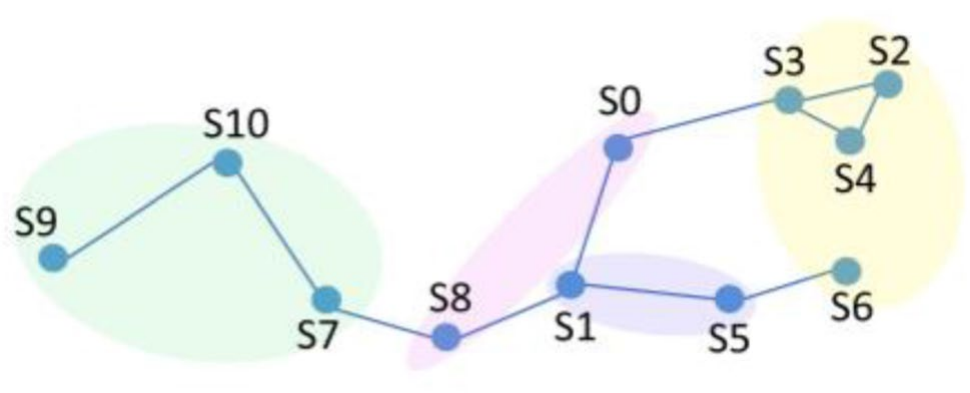
Spatiotemporal similarity distribution map of the monitoring stations in the first quarter.

**Figure 8:**
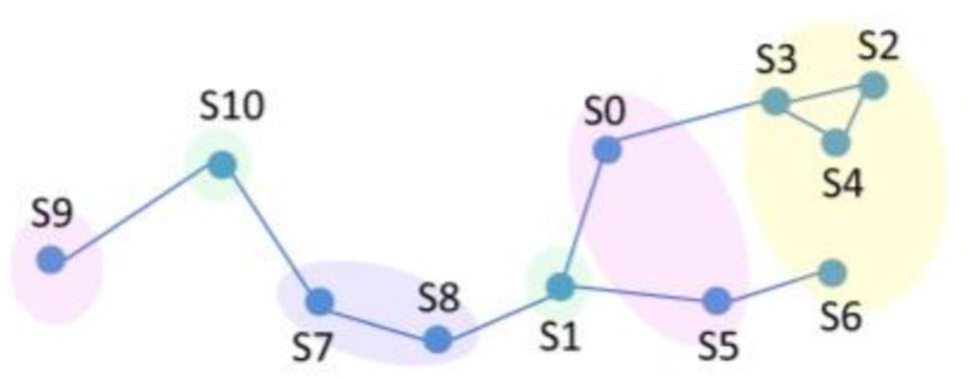
Spatiotemporal similar distribution of monitoring stations in the second and third quarters.

**Figure 9:**
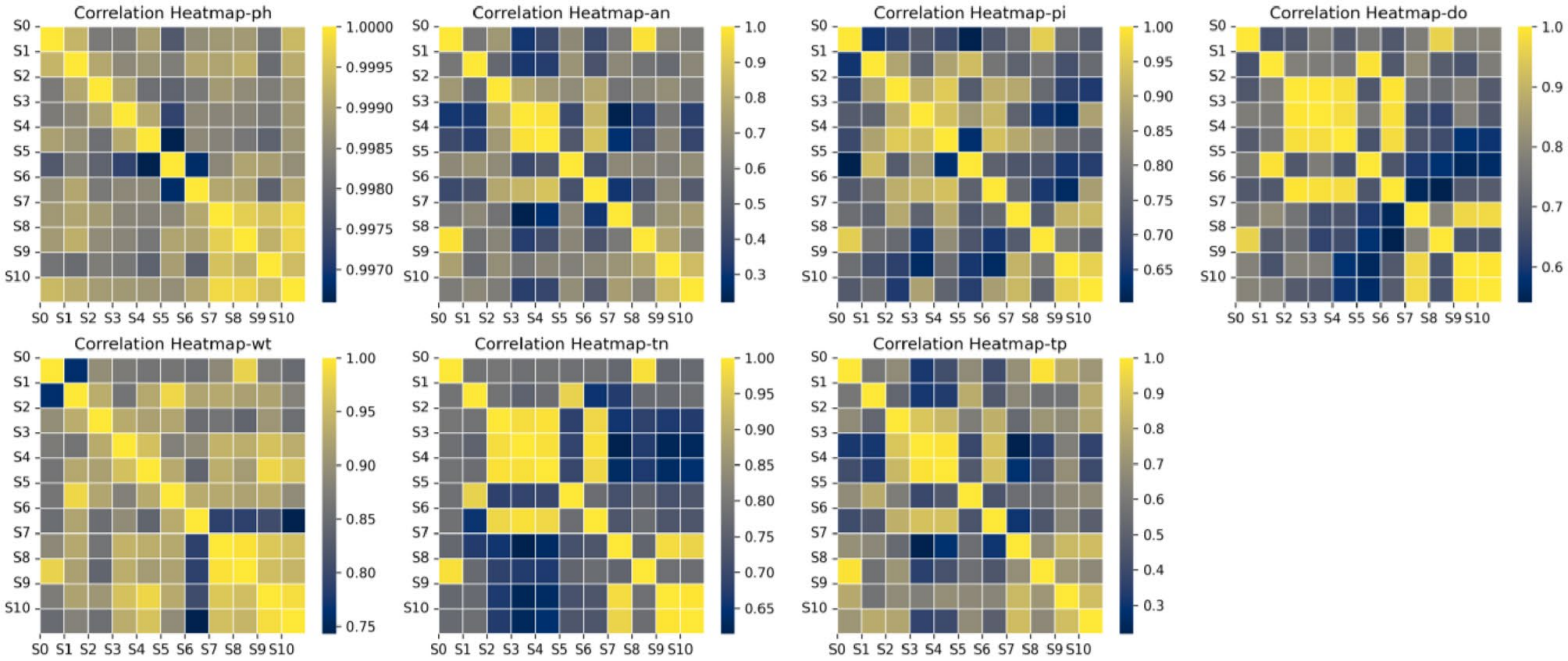
Similarity map of pollutants at monitoring points in the first quarter.

**Figure 10:**
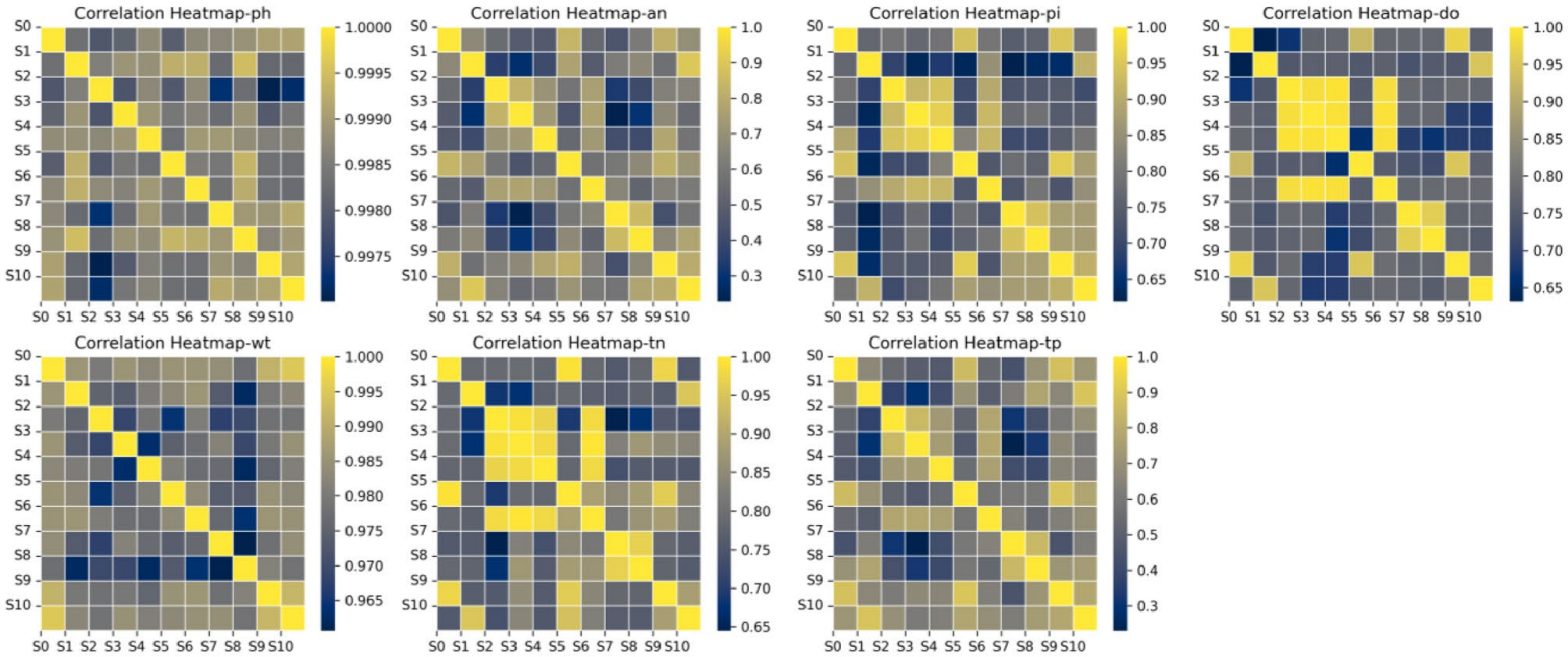
Similarity map of pollutants at monitoring sites in the second and third quarters.

Judging from the above clustering results, each year is divided into three quarters, and different monitoring points can be divided into different areas according to seasons. The spatiotemporal similarity diagrams in different seasons are shown in Figs. 7 and 8. Planes consisting of water quality parameters are very helpful in interpreting the obtained clustering results^38^. According to the water quality correlation results in Figs. 9 and 10, it can be seen that the pollutant monitoring indicators of the four sites S2, S3, S4, and S6 are highly similar. Observing the discharge of monitoring indicators at the site in Table 1, the total phosphorus content is lower and the water quality is better than that of other sites. Moreover, the spatial locations of the four stations are all at the upstream end of the watershed, so they are less polluted. This result may be due to the self-purification effect of the water body. Overall, the water quality at these four sites was good.

The similar points in the first quarter are S0 and S8, S1 and S5, S7 and S9, and S10. It can be seen from the correlation of water quality pollutants that similar sites in the first quarter are highly similar in ammonia nitrogen, total phosphorus, and total nitrogen emissions. Except for S9, the concentration of total phosphorus is relatively high, and the water quality is in a polluted state. This result may be due to the fact that when the basin is in the first quarter, the S0 site is in a densely populated area and S8 is at the river confluence, affected by winter heating and seasonal changes in colored soluble organic matter (CODM) in the water^39^. S1 and S5 are on the same main stream of the river, with slow water flow and similar pollutant distribution. S7, S9, and S10 are in the same main stream, and the pollutant index total nitrogen content is all high. They are less affected by other factors in winter and are all in a high pollution state.

Similar points in the second and third quarters can be divided into three categories, among which S0, S5, and S9 are divided into one category. From the correlation of pollutant distribution, it can be seen that the similarity of pollutants at the sites is high, and the second and third quarters are in the wet season. S0 and S5 are affected by the upstream self-purification sites, and the water quality is relatively improved. This is also reflected in other articles^40^. S1 and S10 are located in the middle of the main stream, with high similarity in pollutant distribution. The two sites have higher total phosphorus and total nitrogen contents, which may be caused by agricultural pollution emissions and chemical plant pollution emissions in summer. S7 and S8 are both located at the end of the main stream and are easily affected by rainfall in summer. Upstream pollution is discharged downstream with the water flow, resulting in high pollutant content at both sites. This result may be due to the fact that heavy metals are more active during the flood season and the site is in a highly polluted state^41^.

### Validation of results

**Figure 11:**
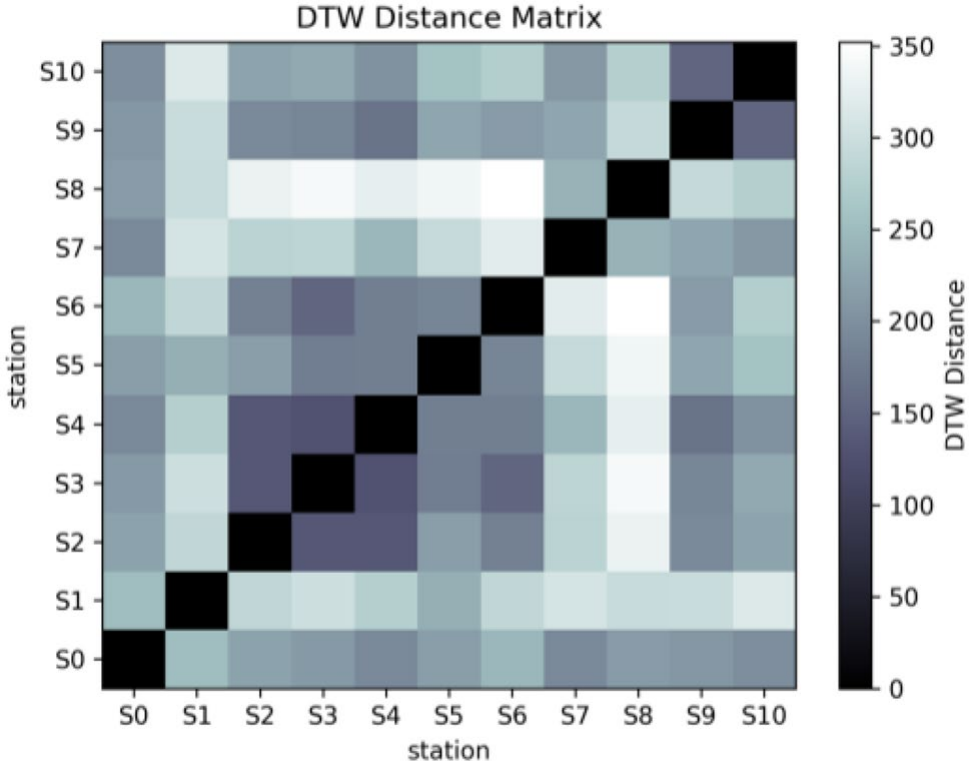
Dynamic time warping distance matrix.

**Figure 12:**
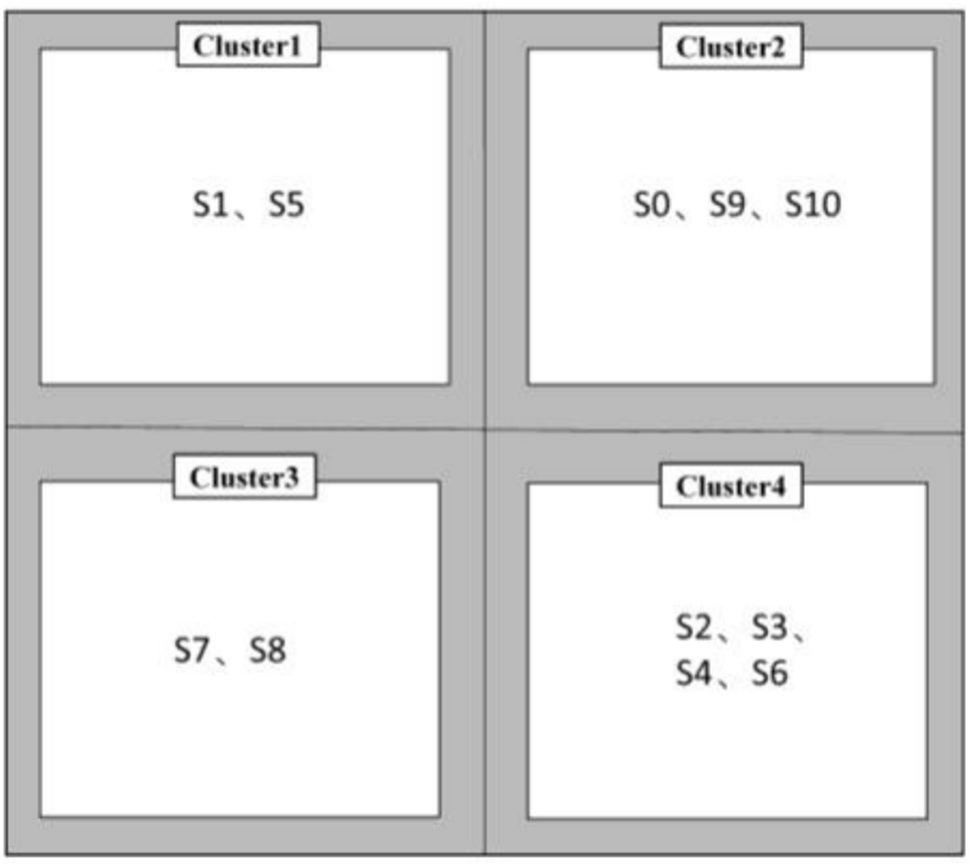
DTW time series clustering results.

Dynamic Time Warping (DTW) is an algorithm used to compare the similarity between two time series. It has been widely used in fields such as time series analysis, speech recognition, and handwriting recognition. The core idea of DTW is to find the best match between two time series while allowing a certain degree of timeline distortion^42^. Therefore, we use this method to classify monitoring points to verify the clustering results of the proposed method. The DTW distance matrix is shown in Fig. 11, and the results of classifying it using the clustering algorithm are shown in Fig. 12. The monitoring sites are divided into four clusters, namely cluster 0 (S1, S5), cluster 1 (S0, S9, S10), cluster 2 (S7 and S8), cluster 3 (S2, S3, S4, S6). However, except for monitoring points with obvious characteristics, other clusters cannot be easily distinguished in the DTW algorithm, and seasonal relationships between sites cannot be easily distinguished. For example, S0, S9 and S10 are clustered together because they have the same time series trend. However, when the seasons are different, the water quality of S9 will be better than that of the other two sites.

## Conclusion

This paper proposes an improved watershed partitioning algorithm that combines the TADW algorithm with the clustering algorithm. In particular, the attribute graph embedding technique embeds the spatial structure and feature-based attributes of different monitoring sites into vectors. In order to obtain the best embedding vector, we use the representative algorithm-TADW. We incorporate the cosine similarity matrix to improve the input features of the TADW algorithm. Through continuous iteration, the improved graph embedding method can extract embedding vectors of different monitoring points. At the same time, the attribute characteristics of different monitoring stations are combined with the embedding vectors to obtain the spatio-temporal feature representation vectors with attribute characteristics of different monitoring stations. In addition, we conducted experiments on a real data set in the Liaohe River Basin and verified the experimental results. The results show the effectiveness of the RTADW clustering method in watershed partitioning. At the same time, we compared the experimental results of DTW and found that our method can better capture the temporal changes of the water ecosystem and have better results in extracting spatio-temporal features. In future work, we will continue to explore the embedded representation of spatiotemporal data and try to build a spatiotemporal combination model. In addition, we can also study other techniques of graph embedding, analyze rich information from other sources, propose new system methods, and apply them to smart water environment protection projects.

## Data Availability

The datasets used and analysed during the current study available from the corresponding author on reasonable request.
